# Non-surgical treatment of peri-implantitis with and without erythritol air-polishing a 12-month randomized controlled trial

**DOI:** 10.1186/s12903-023-02973-5

**Published:** 2023-04-24

**Authors:** Armin Selimović, Dagmar F. Bunæs, Stein Atle Lie, Målfrid Aa. Lobekk, Knut N. Leknes

**Affiliations:** 1 Department of Clinical Dentistry, Faculty of Medicine, University of Bergen, Aarstadveien 19, N-5009 Bergen, Norway; 2 Oris Dental Madla, Stavanger, Norway

**Keywords:** Air-polishing, Erythritol, Peri-implantitis, Implant maintenance, Visual analogue scale

## Abstract

**Background:**

A variety of interventions have been explored in the non-surgical management of peri-implantitis. In spite of extensive testing of various study protocols, effective treatments largely remain unavailable. The objective of the present 12-month single-centre, examiner-masked, randomized controlled clinical trial was to explore whether a low-abrasive erythritol air-polishing system produces added clinical benefit when used adjunctive to conventional non-surgical management of peri-implantitis and to record any associated patient-centered outcomes.

**Methods:**

Forty-three patients with mild to severe peri-implantitis including at least one implant either received ultrasonic/curette subgingival instrumentation and erythritol air-polishing (test) or ultrasonic/curette instrumentation only (control) at baseline and at 3, 6, 9, and 12 months. Probing depth (PD), bleeding on probing (BoP), dental plaque, suppuration (SUP), crestal bone level (CBL), and peri-implant crevicular fluid (PCF) were recorded at baseline, 6 and 12 months. Visual Analogue Scale (VAS) scores were collected immediately following subgingival interventions at all time-points.

**Results:**

A reduction in PD was observed from baseline to 6 months for the test (*p* = 0.006) and control (*p* < 0.001) and from baseline to 12 months for the control (*p* < 0.001). No intergroup differences were observed for primary outcome variables PD or CBL over time (*p* > 0.05). At 6 months, a intergroup difference in PCF was observed in favor of the test (*p* = 0.042). Moreover, a reduction in SUP from baseline to 6 and 12 months was observed in the test (*p* = 0.019). Overall, patients in the control group experienced less pain/discomfort compared with the test (*p* < 0.05), females reporting more pain/discomfort than males (*p* = 0.005).

**Conclusions:**

This study confirms that conventional non-surgical management of peri-implantitis produces limited clinical improvement. It is shown that an erythritol air-polishing system may not produce added clinical benefits when used adjunctive to conventional non-surgical management. In other words, neither approach effectively resolved peri-implantitis. Moreover, the erythritol air-polishing system produced added pain/discomfort particularly in female patients.

**Trial registration:**

The clinical trial was prospectively registered in ClinicalTrials.gov with registration NCT04152668 (05/11/2019).

## Background

Peri-implantitis is characterized as a peri-implant mucosal inflammatory lesion coupled with progressive loss of supporting alveolar bone induced due to local accumulation of microbial biofilm [1, 2]. Early intervention appears critical to regain peri-implant mucosal health and arrest further implant bone loss [2, 3]. Hence, several non-surgical and surgical treatment protocols have been proposed, however, with limited clinical benefit [4, 5]. The primary objective of non-surgical treatment is to facilitate patient daily biofilm removal and professional decontamination of the implant disrupting biofilm formation and removing calculus. As surgical management of peri-implantitis is in general the advocated therapeutic intervention, peri-implantitis patients should be carefully monitored in a supportive care program including professionally administrated biofilm removal. Nevertheless, present non-surgical treatment appear unpredictable with time-limited benefit [6].

Several approaches for implant surface decontamination, including titanium curettes, air abrasive devices, ultrasonic devices, oscillating brushes, and lasers have been evaluated [7]. Air-polishing disrupts biofilm formation producing a fine jet of compressed air, water, and fine powder particles. Recent generations air-polishing devices have been equipped with low-abrasive glycine [8, 9], trehalose [10] or erythritol powders [11–14], coupled with a nozzle for submucosal peri-implant delivery [10, 15–18]. Erythritol is a non-toxic, chemically neutral, and completely water-soluble polyol produced by the reduction of erythrose [19]. The powder is non-caloric, has a high gastrointestinal tolerance, and does not increase blood glucose or insulin levels [20]. The Commercially available erythritol has a mean particle size of 14 µm with low abrasiveness [21]. Studies comparing conventional mechanical debridement with erythritol air-polishing in periodontitis patients, have reported similar results in supportive periodontal therapy (SPT) relative to clinical and microbiological outcomes [18, 22]. Such observations are also reflected in systematic reviews reporting that air-polishing systems as a monotherapy are comparable to conventional therapy in patients undergoing SPT in single- and multi-rooted teeth [23, 24].

In vitro studies may suggest that erythritol appears more effective than previous agents [21, 25]. In addition, the submucosal nozzle extends the application of air-polishing to reach deep submucosal implant sites to disrupt biofilm formation with minimal implant surface distortion [21]. Favorable clinical outcomes of air-polishing powder therapy of peri-implantitis have been observed [26, 27]. Compared with conventional treatment, higher patient satisfaction, improved time efficiency, without adverse events have been reported [28]. However, a recent 12-month randomized controlled trial (RCT), indicated that erythritol air-polishing as monotherapy had limited effect on subgingival decontamination of periodontal furcation defects compared with conventional mechanical debridement [29]. No prospective studies have reported on benefits of repeated submucosal decontamination of peri-implantitis lesions over 12 months using a low-abrasive erythritol air-polishing system as an adjunct to conventional mechanical instrumentation. We hypothesize that repeated submucosal instrumentation with a low abrasive erythritol air-polishing has an adjunctive decontamination effect compared with conventional non-surgical therapy.

Therefore, the primary objective of this RCT is to assess whether erythritol air-polishing produces an adjunctive effect to conventional non-surgical treatment of peri-implantitis in a cohort of maintenance patients, performed every third month over 12 months. A second objective is to evaluate if such adjunctive treatment influences patient comfort.

## Methods

The study protocol and informed consent following the Helsinki Declaration of 1975 (version 2008) was approved by the Medical Research Ethics Committee (2019/30233), University of Bergen, Norway. The clinical trial was prospectively registered in ClinicalTrials.gov with registration NCT04152668 (05/11/2019). The study was conducted as a single-masked RCT. Prior to inclusion, participating patients read and signed the informed consent after the investigators had provided a thorough explanation of the study procedure and its associated risks and benefits. All methods were carried out in accordance with relevant guidelines and regulations. The CONSORT guidelines for reporting RCTs were followed.

### Pre-study calibration and training

Prior to initiation of study, a calibration exercise was performed to document intra-examiner reproducibility for primary outcome variables probing depth (PD) and peri-implant crestal bone level (CBL). In a sample of 10 patients with a total of 19 implants, PD (six sites/implant; mesio-buccal, mid-buccal, disto-buccal, mesio-lingual/palatal, mid-lingual/palatal, disto-lingual/ palatal) and CBL (two sites/implant; mesial and distal on standardized intraoral radiographs) were measured twice at each implant one day apart. PD was measured without removing the prosthetic restoration since both cemented and screw-retained fixed prosthesis were included. Intraclass correlation coefficients (ICCs) were calculated separately for each site. ICC for the agreement ranged between 0.90 and 1.00 for both PD and CBL [30]. The calibration exercise did not include the secondary outcome variable bleeding on probing (BoP).

The operator was trained in the proper use of the latest version of the air-polishing device (AIRFLOW® One, EMS, Nyon, Switzerland) using 10 patients.

### Sample size

In a previous study, sample size estimation based on changes in PD around teeth, a difference of 0.5 mm was considered clinically relevant [31]. Standard deviation of the difference between repeated PD measurements from the intra-calibration exercise was calculated to 0.5 mm. A power analysis based on 43 subjects with the level of significance (α) set to 5%, resulted in 93% power to detect a true difference of 0.5 mm. For the present study dealing with implants, we consider a difference of 1 mm to be clinically relevant, and assume a standard deviation of 1 mm. Further, a 20% drop-out from baseline to follow-up was anticipated. By including 43 patients (23 in test and 20 in control), the calculated statistical power is 89%.

### Study subjects

The study subjects were recruited among patients in maintenance care program in a private dental clinic, Stavanger, Norway, from December 2019 through November 2020. Eligible subjects were healthy adults, age 20–85 years, who had received one or more dental implants that had been in function for more than 12 months and restored with an adequate prosthetic restoration. The inclusion criterium was diagnosis of peri-implantitis on at least one dental implant based on the definition from the consensus report of workgroup 4 of the 2017 World Workshop [32]. Because baseline data were available peri-implantitis diagnosis was based on (I) progressive bone loss beyond initial bone remodeling (CBL loss ≥ 2 mm), (II) increased PD compared with previous examinations in at least one site around the implant (PD ≥ 4 mm), and (III) presence of bleeding and/or suppuration on gentle probing [33] Initial full mouth or experimental site-specific dental plaque scores were not an inclusion criterium. Staging of periodontitis of the included patients was performed according to the 2017 classification [34].

Exclusion criteria were surgical treatment of peri-implantitis within the last 6 months (previous surgical management of peri-implantitis was not an exclusion criterium), inflammation around the implant without evidence of bone loss or with CBL loss < 2 mm, maintenance care therapy within 3 months before initiation of the study, and use of systemic antibiotic within 6 months. Subjects with diabetes mellitus, cancer, HIV/AIDS, acute infections, liver or kidney dysfunction/failure, a history of non-compliant behavior, periapical peri-implantitis, implant fracture, ceramic implants, current pregnancy or breast-feeding, allergy to erythritol, and deficient fixed prosthesis were also excluded [3]. Smoking habits were subjectively reported and registered, but smoking was not an exclusion criterium as smokers are also candidates for dental implants. Smokers were current smoker and non-smokers were never smoker or former smoker who has not smoked in the last 5 years.

### Randomization and treatment

Following baseline examination, included patients were block- randomized to either receive subgingival ultrasonic/curette instrumentation and the erythritol powder/air-polishing system (test) or conventional ultrasonic/curette instrumentation only (control). Block-size for the randomization was set to 10. The computer-generated lists were printed and packed in opaque envelopes marked by randomization numbers. Prior to treatment interventions, the sealed envelopes containing group allocation, were opened by the operator (AS). Author ML, masked to treatment assignments, performed all clinical and radiographic recordings. Operator AS, unaware of previously recorded data, performed peri-implant crevicular fluid (PCF) sampling and all treatments, which were performed at baseline and repeated at 3, 6, 9, and 12 months.

Pre-treatment, each patient rinsed for one minute with a mouthwash, a combination of chlorhexidine and hydrogen peroxide, intended to reduce potential COVID-19 transmission [35]. Implants in both test and control groups were instrumented with titanium curettes (Langer and Langer, Rønvig, Denmark) and an ultrasonic device (Piezotome, Acteon, Bordeaux, France) with a titanium tip (Implant protect kit, Acteon, Bordeaux, France), power set at 75% using water coolant. Care was taken applying the titanium tip with a tangential movement to protect the implant surface structure. Implants in the test group additionally received a low abrasive erythritol powder (Air-flow® Plus, EMS, Nyon, Switzerland; particle size 14 µm) delivered from a PerioFlow® handpiece equipped with an airflow unit (Airflow One®, EMS, Nyon, Switzerland). The handpiece was fitted with a nozzle designed for submucosal peri-implant delivery, directing the air jet perpendicular to the implant surface from the tip of the nozzle (PerioFlow®nozzle, EMS, Nyon, Switzerland). Implants in the control group were polished using polishing paste delivered with a rotating rubber cup. Treatments were carried out without local anesthesia and time limits. Instrumentation ended when the operator considered the implant surfaces free from supra- and submucosal deposits. Treatment times were not recorded.

Following treatment of test and control implants, remaining teeth and implants in the control group were treated with ultrasonic and hand-instruments and polished using polishing paste and rubber cup. In the test group, the remaining teeth and implants were treated with ultrasonic and hand- instruments and the low-abrasive erythritol powder.

Based on the percentage tooth and implant surfaces with visible plaque, the patients received individualized oral hygiene instruction at each appointment. At the end of the study, each patient was re-enrolled in an individually tailored maintenance care program in which site-specific adjunctive therapy including surgical treatment was continuously considered.

### Clinical and radiographic recordings

Prior to treatment, the following clinical parameters were collected at baseline and at 6, 12 months by author ML masked to treatment assignments:

1. PD: distance (mm) from the implant mucosal margin to the probable base of the pocket at six sites per implant (mesio-buccal, buccal, disto-buccal, disto-palatal/lingual, lingual/palatal, mesio-lingual/palatal).
2. BoP: bleeding on gentle probing to the base of the pocket at six sites per implant (mesio-buccal, buccal, disto-buccal, disto-palatal/lingual, lingual/palatal, mesio-lingual/palatal).
3. Full mouth plaque scores recorded as the percentage tooth and implant surfaces with a visible plaque at four sites per tooth/implant (mesial, distal, buccal, and lingual/palatal) following staining with disclosing solution. As a supplement, the probe was used to record presence or absence of plaque at test and control sites.
4. Width of keratinized mucosa at baseline as the distance (mm) from the mid-buccal mucosal margin to the mucogingival junction at each implant.
5. Presence of suppuration (SUP) after gentle probing to the base of the pocket at six sites per implant (mesio-buccal, buccal, distobuccal, disto-palatal/lingual, lingual/palatal, mesio-lingual/palatal.

Clinical recordings were performed using a plastic replacement tip (Colorvue™ Probe, Hu-Friedy, Chicago, IL, USA) with 3–6-9–12 mm markings connected to a satin surfaced steel handle (Colorvue™ Prote Handle (PH6), Hu-Friedy, IL, USA). CBL was recorded from intraoral digitalized radiographs captured using customized radiographic holders (Eggen-holder) and long-cone parallel technique [36]. The Eggen-holder was customized applying a polyvinyl siloxane impression material. Radiographic images were considered suitable for measurements if the whole implant was included in the image. CBL was defined as the distance (mm) between the implant shoulder and the first bone-implant contact (BIC). The distance from the implant shoulder (platform) to the mesial and distal BIC was measured by using a software image-processing program (DIGORA, Soredex, Helsinki, Finland). Known dimensions of the implant (length and diameter) were used to scale each radiographic image and thus allow accurate measurements [37]. The radiographic recordings were performed under 7X magnification [38].

### Crevicular fluid recordings

Peri-implant crevicular fluid (PCF) was collected at baseline, 6, and 12 months [39]. Briefly, sample sites were isolated with cotton rolls, carefully cleaned for supragingival plaque, and air-dried. A perio paper strip was then gently placed 1–2 mm inside the orifice of the site and left in place for 30 s. Next, the perio strip was placed into the Periotron 8000® (Harco, Tustin, CA, USA) previously calibrated to estimate the volume of PCF collected.

### Patient-centered outcomes

VAS recordings were used to estimate postoperative patient pain/discomfort during test and control treatment [40, 41]. Scorings were performed at baseline and at 3, 6, and 12 months post-instrumentation immediately following completion of instrumentation on a horizontal line measuring 100 mm, scores 0 = “*completely dissatisfied/painful/uncomfortable*” and 10 = “*completely satisfied/without pain/comfortable*” [42]. Prior to therapy, patients were queried about pain to rule out pre-existing pain/discomfort unrelated to study treatments.

Two different VAS scores were registered separately for the following questions:

- VAS1: How satisfied are you with today’s treatment?
- VAS2: Did you experience the treatment as painful/uncomfortable?

### Statistical analysis

Data was entered into MS-Excel, proofed for entry errors, and imported into Stata (Stata version 17, StataCorp LLC, College Station, TX, USA). All data analyses were performed by a statistician (SAL) at Bergen University who had not participated in data collection or processing. Primary outcome variables were PD (mean of six sites/implant) and CBL loss (mean of mesial and distal recordings). Plaque, BoP, SUP, PCF, and VAS scores were defined as secondary outcome variables. Additionally, PD was dichotomized as < 6 mm and ≥ 6 mm and analyzed as a categorical variable.

Descriptive statistics were calculated separately for the two groups at different time points at baseline, and at 6 and 12 months. Chi-square tests were used to identify differences for categorical variables at baseline, while t-tests were applied for continuous variables. To test changes over time and differences between groups, adjusted multivariable regression analyses, including robust variance estimates to adjust for repeated (multiple) observations for each individual, were applied. The continuous outcomes were analyzed using linear models, while categorical variables were analyzed using logistic regression models. Regression analyses including other potential risk factors were performed to explore if any of these predicted the outcome variables. P-values less than 0.05 were considered statistically significant.

## Results

**Fig. 1:**
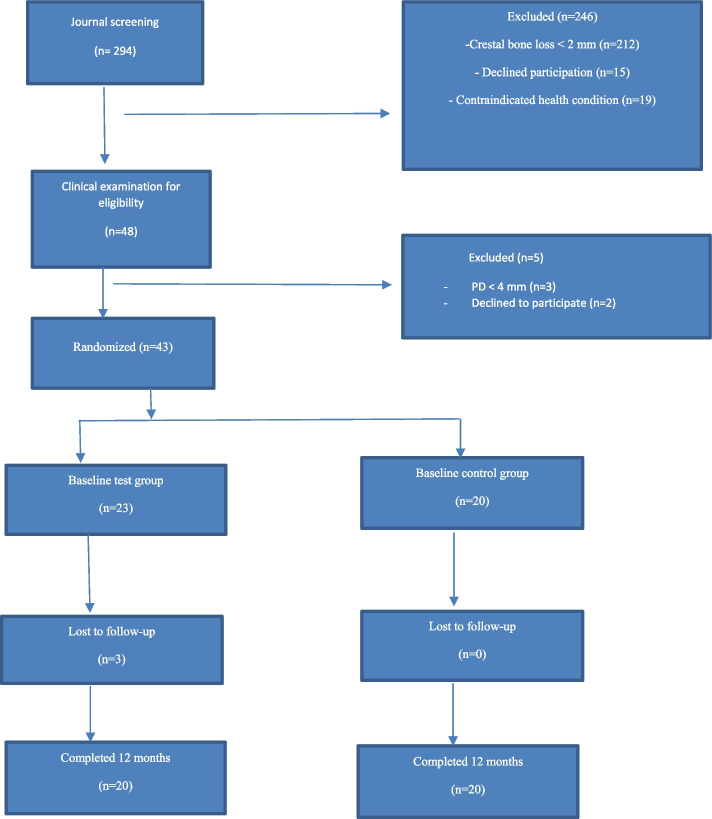
Flowchart of study

Of 294 patient charts that were screened from October 2019 to May 2020, 48 met the inclusion criteria and were clinically examined (Fig. 1). Three patients were excluded during the clinical examination due to PD < 4 mm (i.e., a reduction in PD compared with previous records) and two patients declined to participate. Of the 43 patients included (62 implants), 23 were block randomized to the test group and 20 to the control group. In total, 40 patients (57 implants) completed the 12-month study and were included in the statistical analysis. Two patients (three implants) were excluded due to implant fracture and mobility by the 3-month examination. One patient with two implants declined to return to follow-up visits due to cost. No adverse events were reported. The mean age of the study group was 65.1 years, range 33–83 years. Details of age, gender, smoking habits, stage of periodontitis, implant location, brand, years in function, implant length, width of keratinized mucosa, and type of crown/bridge connection of the test and control groups respectively are specified in Table1. There were no intergroup differences at baseline for any of these variables.

**Table 1:** Patient and implant characteristics

**Variable**	**Test *n* = 23 (31 implants)**	**Control *n* = 20 (31 implants)**	*P*
Mean age, years	65,8 ± 11.6	64,5 ± 13.6	0.722
Sex			0.172
Female	14 (61%)	8 (40%)	
Male	9 (39%)	12 (60%)	
Smoking			0.541
Smoker	4 (17%)	5 (25%)	
Non-smoker	19 (83%)	15 (75%)	
Stage of periodontitis			0.191
Stage I/Stage II	7 (30%)	10 (55%)	
Stage III/Stage IV	16 (70%)	10 (45%)	
Location			0.224
Maxilla	22 (71%)	26 (84%)	
Mandibula	9 (29%)	5 (16%)	
Brand			0.412
Straumann	12 (39%)	8 (26%)	
Brånemark/Nobel Biocare	17 (55%)	22 (71%)	
Another system	2 (6%)	1 (3%)	
Years in function			0.392
< 5 years	6 (20%)	2 (7%)	
5 – 10 years	8 (26%)	9 (29%)	
10 – 15 years	7 (22%)	11(35%)	
> 15 years	10 (32%)	9 (29%)	
Implant length			0.554
< 10 mm	1 (3%)	2 (7%)	
≥ 10 mm	30 (97%)	29 (93%)	0.576
Keratinized mucosa width			
< 2 mm	10 (32%)	8 (26%)	
≥ 2 mm	21 (68%)	23 (74%)	
Crown/bridge connection			0.587
Screw-retained	20 (65%)	22 (71%)	
Cemented	11 (35%)	9 (29%)	

### Clinical outcomes

**Fig. 2:**
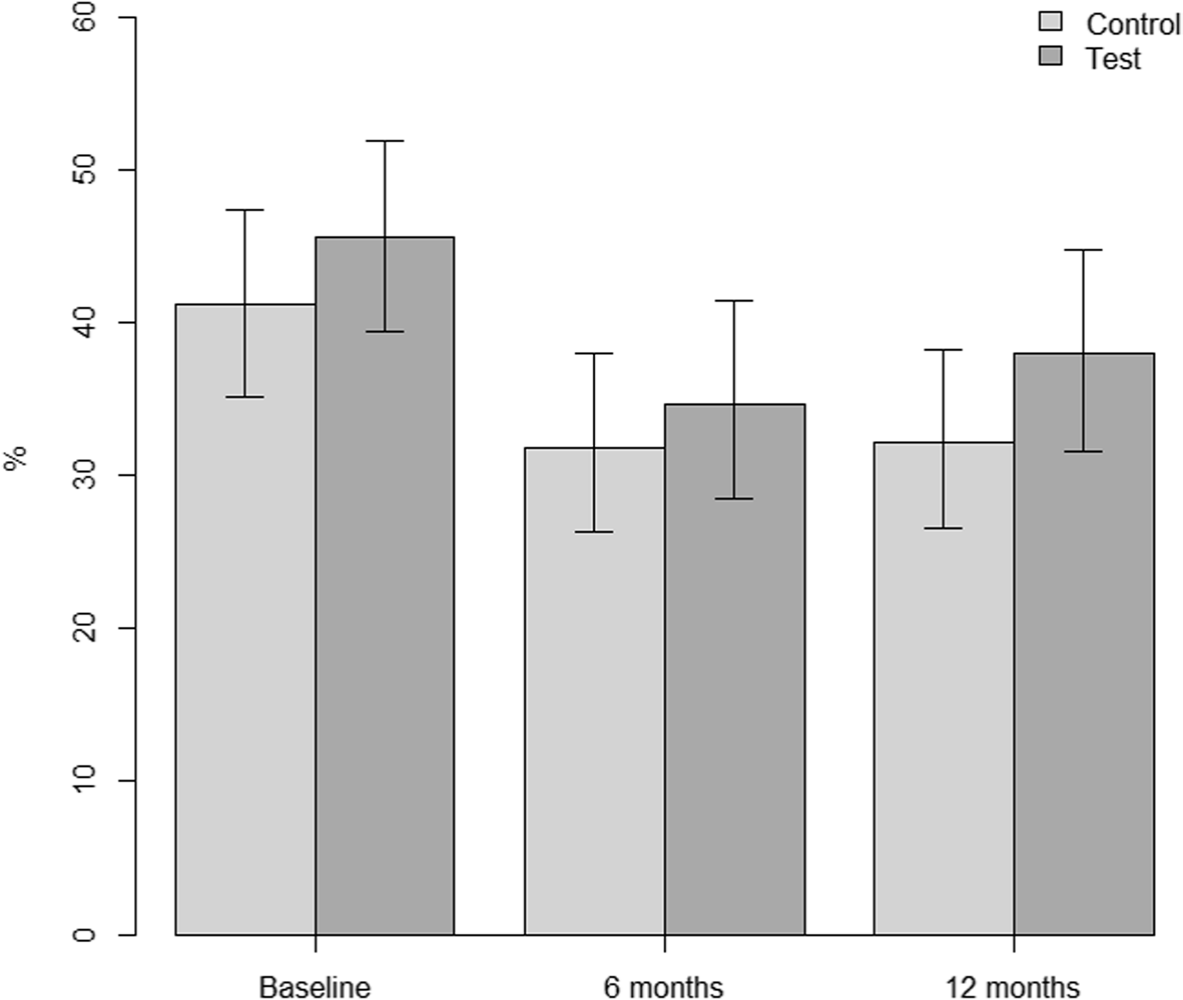
Proportion of sites ≥ 6 mm for the test and control group at baseline, 6 months, and 12 months

Table 2 depicts between-group differences and changes over time at implant level. Mean PD was significantly reduced between baseline and 6 months for both groups (test, *p* = 0.006, control, *p* < 0.001), but not between 6 and 12 months. Between baseline and 12 months, only mean PD for control was significantly reduced (*p* < 0.001). Between baseline and 12 months, the reduction in mean PD for control was significantly larger than for test (-0.6 and -0.3, respectively; *p* = 0.029). For the proportion of sites with PD ≥ 6 mm, a statistically significant decrease was detected over time for both groups from baseline to 6 months (control 41.1% to 31.9%; *p* = 0.001; test 45.6% to 34.6%; *p* < 0.001) and from baseline to 12 months (control 41.1% to 32.1%; *p* = 0.011; test 45.6% to 38.0; p = 0.002). No overall difference in the change between the groups was observed (*p* = 0.775; Fig. 2). No significant changes in mean CBL loss were observed over time for either group or between groups (all *p* > 0.05).

**Table 2:** Implant level recordings at baseline, and at 6 months and 12 months and change (Δ) from baseline and 6 months for clinical parameters

	**Baseline**	***P***	**6 months**	***P***	**12 months**	***P***	**Δ**_**0-6**_	***P***	**Δ**_**6-12**_	***P***	**Δ**_**0-12**_	***P***
**Mean PD**												
**Test**	4.5 ± 0.10		4.1 ± 0.10		4.2 ± 0.11		-0.4 ± 0.12	**0.006**	0.1 ± 0.10	0.415	-0.3 ± 0.13	0.107
**Control**	4.4 ± 0.10		3.9 ± 0.09		3.8 ± 0.09		-0.5 ± 0.09	**< 0.001**	-0.1 ± 0.08	0.702	-0.6 ± 0.07	**< 0.001**
**Difference**	0.1 ± 0.22	0.704	0.2 ± 0.24	0.410	0.4 ± 0.27	0.138	0.1 ± 0.14	0.428	0.2 ± 0.13	0.116	0.3 ± 0.14	**0.029**
**Mean CBL**												
**Test**	3.6 ± 0.22		3.3 ± 0.18		3.5 ± 0.21		-0.3 ± 0.28	0.611	-0.1 ± 0.08	0.328	-0.2 ± 0.30	0.881
**Control**	3.1 ± 0.20		3.0 ± 0.23		3.4 ± 0.27		-0.1 ± 0.21	0.861	0.4 ± 0.21	0.186	0.3 ± 0.16	0.259
**Difference**	0.5 ± 0.46	0.320	0.3 ± 0.43	0.488	-0.0 ± 0.52	0.960	-0.2 ± 0.35	0.648	-0.3 ± 0.22	0.222	-0.4 ± 0.34	0.210
**PCF**												
**Test**	89.5 ± 5.8		46.5 ± 5.2		51.1 ± 5.8		-43.0 ± 4.6	** < 0.001**	4.6 ± 4.7	0.627	-38.3 ± 6.7	**< 0.001**
**Control**	76.3 ± 5.9		49.7 ± 6.0		55.7 ± 5.9		-26.6 ± 6.4	**0.002**	6.0 ± 4.7	0.463	-20.6 ± 4.9	**0.002**
**Difference**	13.1 ± 8.6	0.134	-3.2 ± 9.2	0.727	-4.6 ± 10.1	0.650	-16.4 ± 7.8	**0.042**	-1.4 ± 6.6	0.835	-17.8 ± 8.2	**0.037**
							OR_0-6_		OR_6-12_		OR_0-12_	
**Plaque (%)**												
**Test**	45.2		19.2		34.6		0.29	0.125	2.22	0.535	0.64	0.753
**Control**	54.8		38.7		45.2		0.52	0.554	1.30	0.927	0.68	0.763
**Odds ratio (OR)**	0.68	0.508	0.38	0.188	0.64	0.479	0.56	0.487	1.71	0.584	0.95	0.945
**BoP (%)**												
**Test**	59.7		37.8		36.5		0.41	**< 0.001**	0.95	0.947	0.39	**< 0.001**
**Control**	58.1		32.3		32.3		0.34	**< 0.001**	1.00	0.999	0.34	**< 0.001**
**Odds ratio (OR)**	1.07	0.807	1.28	0.455	1.20	0.480	1.19	0.522	0.95	0.825	1.13	0.637
**SUP (%)**												
**Test**	32.3		7.7		7.7		0.18	**0.019**	1.00	0.999	0.18	**0.019**
**Control**	16.7		6.5		6.5		0.34	0.210	1.00	0.999	0.34	0.210
**Odds ratio (OR)**	2.38	0.218	1.21	0.858	1.21	0.858	0.51	0.427	1.00	0.999	0.51	0.427

PD Probing depth, *CBL* Crestal bone level in mm, and *PCF* Peri-implant crevicular fluid in µL are presented as mean values and SEM, while Plaque, *BoP* Bleeding on probing, and *SUP* Suppuration as percentages and *OR* Odds ratios

Mean PCF volume decreased significantly between baseline and 6 months for both groups (test, *p* < 0.001; control, *p* = 0.002). Similar significant decreases were observed between baseline and 12 months (test, *p* < 0.001; control, *p* = 0.002). Between baseline and 6 months and baseline and 12 months, reductions in PCF for test were significantly larger than for control (-43.0 and -26.6-; *p* = 0.042:-38.3 and -20.6; *p* = 0.037, respectively).

For visible plaque, no changes over time were observed for either group (all *p* > 0.05). In contrast, the proportion of BoP was significantly reduced between baseline and 6 months and between baseline and 12 months for both groups (all *p* < 0.001). The proportion of SUP was significantly reduced between baseline and 6 months and between baseline and 12 months for test only (both, *p* = 0.019).

### Patient pain/discomfort outcomes

Following instrumentation, no intra- or intergroup differences were observed regarding satisfaction with treatment (VAS1) at any observation interval (Table 3). Pain/discomfort (VAS2) scores at baseline, 3, 6, and 9 months were all significantly elevated (less pain/discomfort) for control compared with the test (p-values ranging from 0.005 to 0.040), no group differences were discerned at 12 months. Between 6 and 12 month and baseline and 12 months, VAS2-values showed a greater increase for the test than for the control (0.79 and -0.33; *p* = 0.008: 1.39 and -0.39; *p* = 0.005, respectively). The regression analysis revealed that females reported more pain and discomfort (*p* = 0.005).

**Table 3:** Patient mean scores (± SEM) for Visual Analogue Scale (VAS) 1 and 2 at baseline and 3, 6, 9, and 12 months and mean changes (Δ) from baseline and 6 months

	**Baseline**	***p***	**3 Months**	***p***	**6 Months**	***p***	**9 Months**	***p***	**12 Months**	***p***	**Δ**_**0-6**_		**Δ**_**6-12**_		**Δ**_**0-12**_	
	Mean ± SEM		Mean ± SEM		Mean ± SEM		Mean ± SEM		Mean ± SEM		Mean ± SEM	*p*	Mean ± SEM	*p*	Mean ± SEM	*p*
**VAS 1**																
**Test**	8.8 ± 0.49		9.3 ± 0.15		9.3 ± 0.16		9.3 ± 0.13		9.6 ± 0.11		0.52 ± 0.52	0.906	0.23 ± 0.13	0.568	-0.75 ± 0.50	0.695
**Control**	9.4 ± 0.15		9.5 ± 0.10		9.5 ± 0.16		9.6 ± 0.10		9.5 ± 0.14		0.10 ± 0.11	0.933	0.25 ± 0.17	0.999	0.12 ± 0.16	0.963
**Difference**	-0.6 ± 0.51	0.260	-0.2 ± 0.18	0.405	-0.2 ± 0.23	0.509	-0.3 ± 0.17	0.113	0.1 ± 0.18	0.767	0.43 ± 0.53	0.423	0.20 ± 0.22	0.345	0.63 ± 0.52	0.233
**VAS 2**																
**Test**	7.4 ± 0.56		8.1 ± 0.41		8.1 ± 0.33		8.0 ± 0.33		8.8 ± 0.25		-0.61 ± 0.52	0.843	0.79 ± 0.31	0.208	1.39 ± 0.53	0.181
**Control**	9.1 ± 0.20		9.1 ± 0.20		9.0 ± 0.24		9.1 ± 0.20		8.7 ± 0.21		0.07 ± 0.20	0.998	-0.33 ± 0.26	0.812	-0.39 ± 0.28	0.758
**Difference**	-1.7 ± 0.59	0.006	-1.0 ± 0.46	0.040	-1.0 ± 0.41	0.018	-1.0 ± 0.36	0.009	0.09 ± 0.33	0.785	0.67 ± 0.55	0.225	1.10 ± 0.40	0.008	1.78 ± 0.60	0.005

## Discussion

The objective of this 12-month, examiner-masked, randomized, controlled clinical trial was to explore whether a low-abrasive erythritol air-polishing system offers adjunctive clinical effects to conventional non-surgical treatment of peri-implantitis, and secondly, to evaluate patient-centred outcomes using VAS. In short, the observations herein indicate that both treatment approaches support clinical improvements. For both groups, a significant reduction in PD was observed from baseline to 6 months, but at 12 months only for the control group. CBLs showed no change over time for either group. BoP was significantly reduced from baseline to 12 months for both treatments. Following instrumentation, less discomfort and pain was reported from the control group and significantly less by males.

At implant sites with peri-implantitis, PD is correlated with bone loss and therefore serves as a relevant surrogate for disease severity [32]. PD recordings around implants are influenced by probing errors [43], grade of inflammation [44], PD severity, and probing force [45]. Further, PD recordings around implants with peri-implantitis might be unprecise since the supracrestal collagen fibers are oriented parallel to the implant surface, allowing the probe to penetrate close to alveolar bone [45]. Despite potential confounding factors, increased PD compared with previous recordings comprise part of the World Workshop case definitions of peri-implantitis [32]. Therefore, we defined PD in addition to CBL loss beyond initial bone remodeling, as primary outcome variables.

Both test and control sites showed significant reduction in PD from baseline to 6 months, but at 12 months only for control sites with significant between-group differences. To date, no studies have reported on erythritol air-polishing as an adjunctive therapy to conventional non-surgical treatment of peri-implantitis in a cohort of maintenance patients. A recent RCT, comparing erythritol air-polishing as monotherapy with piezoelectric ultrasonic scaling in non-surgical treatment of peri-implantitis, reported no significant intergroup treatment differences for clinical peri-implant parameters as PD, suppuration on probing, and plaque score at a 3-month observation [26]. Fourteen patients, four in the airpolishing group and 10 in the ultrasonic group were followed over 12 months. Reported 3-month observations are partly congruent with our 6-month findings revealing no intergroup differences. The exact reason for observing a significant intergroup difference at 12 months and not at 6 month in the present study is hard to pinpoint possibly reflecting a pronounced inflammatory response in the test group expressed as a higher percentage BoP and SUP [46].

At baseline the proportions of sites with PDs ≥ 6 was within both groups above 40%. Over time a significant reduction was observed at 6 months followed by a slight relapse at 12 months particularly in the test group (from 35 to 38%). These findings together with rather high BoP at 12 months (test 37% and control 32%) underline the limitations of non-surgical interventions and that neither treatment approach effectively resolve peri-implantitis. Our observations are thus in support of retrospective [5] and prospective [26] analysis concluding that surgical treatment might be indicated for a significant number of peri-implantitis patients. CBL stability is essential for defining peri-implant health and predicting disease progression [32, 47]. The present study revealed that CBL was stable over time, thus demonstrating that conventional mechanical instrumentation with or without erythritol air-polishing may successfully preserve CBL during 12-month of supportive implant therapy. Similar findings are reported by others [48], indicating that none of the non-surgical decontamination approaches of peri-implantitis are preferred.

In the present study, both interventions induced a significant reduction in the inflammatory surrogates. Improvement in secondary outcomes BoP and PCF have been reported previously comparing air-abrasives devices and conventional mechanical debridement [9, 49, 50]. Whereas present study showed a significant reduction in BoP for both groups, they found a significantly greater reduction in BoP in the air-abrasive group at 3 and 6 months concluding that neither control (ultrasonic) nor test (air-abrasive device) may effectively resolve peri-implantitis [26]. PCF provides an early indication of patients at risk for peri-implantitis and implant loss [51, 52]. Inflammatory exudate leaking out into PCF reflects the nature of the inflammatory lesion [53, 54]. In the present study, PCF was significantly reduced for both groups, between baseline and 6 months and between baseline and 12 months. At both observation intervals, the reductions were increased in the test group compared with the control. However, there were no changes in PCF for either group between 6 and 12 months, corroborating the notion that non-surgical treatment of peri-implantitis is unpredictable [6]. A retrospective study of 304 implants with peri-implantitis reported the prevalence of SUP to be 28% at implant level, and that grades of SUP were associated with peri-implant bone loss and increased PD [46]. Increased tissue inflammation may influence healing response, and implants diagnosed with SUP are more frequently associated with a distinct microbiome compared with implants without [55].

Non-surgical dental implant biofilm disruption appears insufficient intervention in patients with less than optimal oral hygiene [56]. In the present study, at 12 months, plaque scores averaged 35% and 45% for the test and control, respectively. All patients had a previous history of regular maintenance and the implants had been exposed to numerous instrumentations. Less than ideal compliance may thus be explained by loss of motivation after years of comprehensive periodontal and implant therapy. Also, it appears more challenging to obtain clinical resolution in residual than in previously untreated lesions [22, 29]. Further, nine patients (22,5%) were smokers with long-term negative effect on mucosal, periodontal, and peri-implant soft-tissue inflammation and loss of CBL [57].

Previous reports showing enhanced effects of air-abrasive treatment have been limited to teeth [12, 18]. One in vitro study showed that erythritol may alter the microstructure and metabolomic profile of the biofilm composed of Streptococcus gordonii and Porphyromonas gingivalis [58]. Moreover, a more effective biofilm reduction in addition to bactericidal effects of erythritol on pathogenic bacteria have also been observed [59–61]. Due to the complexity of the dental implant micro/macro structure, the disruption of the biofilm over the exposed threads might be insufficient for clinical resolution and demands further study to optimize clinical protocol, delivery system, as well as agents for submucosal delivery. The same challenge applies also for non-surgical instrumentation with titanium curettes. In agreement with a systematic review and meta-analysis [62], despite clinical improvements, a complete resolution of inflammation was not achieved by any of the treatment approaches.

For both groups, minor pain/discomfort (VAS2) was reported, with significantly less pain/discomfort experienced in control group at baseline, 3, 6, and 9 months. The difference vanished at 12 months, as after 6 months the VAS scores for the test group decreased whereas continued to increase in the control. In the test group the percentage of implants diagnosed with SUP at baseline was 32.3, whereas the corresponding percentage in the control group was 16.7. Thus, a higher proportion of SUP at baseline in the test group, possibly reflecting a higher degree of peri-implant mucosal inflammation, may partly explain the increased pain/discomfort reported up to 9 months in this group. A previous RCT also reported on pain/discomfort following air polishing and ultrasonic scaling [26], however, a closer between studies comparison is prohibited due to that in this RCT local anesthesia was used as needed during interventions. In the present study, females perceived more pain and discomfort than males also reported by Seymour et al. finding females experiencing more post-operative pain than males [63].

### Strength and limitations

This study was conducted as an RCT. A pre-study calibration exercise and proper training in the use of the latest version of the air-polishing device were performed. The statistical power of study was estimated to 89%. Included patients were block randomized to either test or control treatments to ensure an equal number of patients in each group prohibiting the occurrence of systemic differences between treatment groups [64]. Patient and implant characteristics at baseline were balanced as no significant between-group differences were detected. All patients were re-treated at 3-month intervals ensuring compliance with oral hygiene regime and regular treatments.

We acknowledge that the present RCT is not exempt from limitations. PD was measured without removing the prosthetic restoration since both cemented and screw-retained fixed prosthesis were included. This may affect clinical assessments, especially in the posterior area [65]. No microbiological samplings were performed, and inflammatory PCF markers were not qualitatively analyzed. High quality clinical studies have shown that the biofilm in peri-implantitis contains a more complex microbial composition compared to periodontitis and several bacteria species are identified as peri-implantitis specific pathogens [66]. Further, no true control group was included. However, to date no non-surgical protocol seems to be the gold standard in the treatment of peri-implantitis.

## Conclusions and clinical implications

The observations reported indicate that ultrasonic/curette subgingival instrumentation with and without erythritol air-polishing support clinical improvements. Over a 12-month period, both treatments prevented further bone loss, but did not adequately arrest inflammation. Thus, neither treatment approach effectively resolves peri-implantitis. A significant inter-treatment difference in PD was observed in favor of ultrasonic/curette instrumentation over 12 months. Following instrumentation, less discomfort and pain were reported in the control group. Future RCTs based on more powerful datasets and with longer follow-up periods are needed to draw clear conclusion about the efficiency of erythritol air-polishing alone or in combination with other non-surgical therapies. Moreover, in advance identification of potential patients characteristics increasing the probability of successful non-surgical peri-implantitis therapy, would be of immense importance. Overall, the results highlight a demand for effective non-surgical protocols for decontamination of dental implants diagnosed with peri-implantitis and that surgical management might be indicated for a significant number of peri-implantitis patients.

## Data Availability

The datasets used and analyzed during the current study and coding are available from the corresponding author on reasonable request.

## Abbreviations

VAS: Visual analogue scale
PD: Probing depth
BoP: Bleeding on probing
SUP: Suppuration
CBL: Crestal bone level
PCF: Peri-implant crevicular fluid
RCT: Randomized controlled trial
ICC: Intraclass correlation coefficients

## References

[1] Schwarz F, Derks J, Monje A, Wang HL (2018). Peri-implantitis. J Periodontol.

[2] Salvi GE, Cosgarea R, Sculean A (2017). Prevalence and mechanisms of peri-implant diseases. J Dent Res.

[3] Majzoub J, Chen Z, Saleh I, Askar H, Wang HL (2021). Influence of restorative design on the progression of peri-implant bone loss: a retrospective study. J Periodontol.

[4] Heitz-Mayfield LJ, Mombelli A (2014). The therapy of peri-implantitis: a systematic review. Int J Oral Maxillofac Implants.

[5] Karlsson K, Derks J, Hakansson J, Wennstrom JL, Petzold M, Berglundh T (2019). Interventions for peri-implantitis and their effects on further bone loss: a retrospective analysis of a registry-based cohort. J Clin Periodontol.

[6] Renvert S, Roos-Jansåker AM, Claffey N (2008). Non-surgical treatment of peri-implant mucositis and peri-implantitis: a literature review. J Clin Periodontol.

[7] Figuero E, Graziani F, Sanz I, Herrera D, Sanz M (2014). Management of peri-implant mucositis and peri-implantitis. Periodontol 2000.

[8] Flemmig TF, Hetzel M, Topoll H, Gerss J, Haeberlein I, Petersilka G (2007). Subgingival debridement efficacy of glycine powder air polishing. J Periodontol.

[9] Sahm N, Becker J, Santel T, Schwarz F (2011). Non-surgical treatment of peri-implantitis using an air-abrasive device or mechanical debridement and local application of chlorhexidine: a prospective, randomized, controlled clinical study. J Clin Periodontol.

[10] Kruse AB, Akakpo DL, Maamar R, Woelber JP, Al-Ahmad A, Vach K, Ratka-Krueger P (2019). Trehalose powder for subgingival air-polishing during periodontal maintenance therapy: a randomized controlled trial. J Periodontol.

[11] Hägi TT, Hofmänner P, Salvi GE, Ramseier CA, Sculean A (2013). Clinical outcomes following subgingival application of a novel erythritol powder by means of air polishing in supportive periodontal therapy: a randomized, controlled clinical study. Quintessence Int.

[12] Sultan DA, Hill RG, Gillam DG. Air-Polishing in Subgingival Root Debridement: A Critical Literature Review. J Dent Oral Biol. 2017;2(10):1065.

[13] Mensi M, Scotti E, Calza S, Pilloni A, Grusovin MG, Mongardini C (2017). A new multiple anti-infective non-surgical therapy in the treatment of peri-implantitis: a case series. Minerva Stomatol.

[14] Jentsch HFR, Flechsig C, Kette B, Eick S (2020). Adjunctive air-polishing with erythritol in nonsurgical periodontal therapy: a randomized clinical trial. BMC Oral Health.

[15] Petersilka GJ, Tunkel J, Barakos K, Heinecke A, Häberlein I, Flemmig TF (2003). Subgingival plaque removal at interdental sites using a low-abrasive air polishing powder. J Periodontol.

[16] Petersilka GJ (2011). Subgingival air-polishing in the treatment of periodontal biofilm infections. Periodontol.

[17] Kargas K, Tsalikis L, Sakellari D, Menexes G, Konstantinidis A (2015). Pilot study on the clinical and microbiological effect of subgingival glycine powder air polishing using a cannula-like jet. Int J Dent Hyg.

[18] Hägi TT, Hofmänner P, Eick S, Donnet M, Salvi GE, Sculean A, Ramseier CA (2015). The effects of erythritol air-polishing powder on microbiologic and clinical outcomes during supportive periodontal therapy: Six-month results of a randomized controlled clinical trial. Quintessence Int.

[19] Moon HJ, Jeya M, Kim IW, Lee JK (2010). Biotechnological production of erythritol and its applications. Appl Microbiol Biotechnol.

[20] de Cock P (2018). Erythritol Functional Roles in Oral-Systemic Health. Adv Dent Res.

[21] Drago L, Del Fabbro M, Bortolin M, Vassena C, De Vecchi E, Taschieri S (2014). Biofilm removal and antimicrobial activity of two different air-polishing powders: an in vitro study. J Periodontol.

[22] Muller N, Moene R, Cancela JA, Mombelli A (2014). Subgingival air-polishing with erythritol during periodontal maintenance: randomized clinical trial of twelve months. J Clin Periodontol.

[23] Ng E, Byun R, Spahr A, Divnic-Resnik T (2018). The efficacy of air polishing devices in supportive periodontal therapy: A systematic review and meta-analysis. Quintessence Int.

[24] Onisor F, Mester A, Mancini L, Voina-Tonea A (2022). Effectiveness and clinical performance of erythritol air-polishing in non-surgical periodontal therapy: a systematic review of randomized clinical trials. Medicina (Kaunas).

[25] Moharrami M, Perrotti V, Iaculli F, Love RM, Quaranta A (2019). Effects of air abrasive decontamination on titanium surfaces: A systematic review of in vitro studies. Clin Implant Dent Relat Res.

[26] Hentenaar DFM, De Waal YCM, Stewart RE, Van Winkelhoff AJ, Meijer HJA, Raghoebar GM (2021). Erythritol airpolishing in the non-surgical treatment of peri-implantitis: A randomized controlled trial. Clin Oral Implants Res.

[27] Tastepe CS, van Waas R, Liu Y, Wismeijer D (2012). Air powder abrasive treatment as an implant surface cleaning method: a literature review. Int J Oral Maxillofac Implants.

[28] Moëne R, Décaillet F, Andersen E, Mombelli A (2010). Subgingival plaque removal using a new air-polishing device. J Periodontol.

[29] Ulvik IM, Saethre T, Bunaes DF, Lie SA, Enersen M, Leknes KN (2021). A 12-month randomized controlled trial evaluating erythritol air-polishing versus curette/ultrasonic debridement of mandibular furcations in supportive periodontal therapy. BMC Oral Health.

[30] Landis JR, Koch GG (1977). The measurement of observer agreement for categorical data. Biometrics.

[31] Bunaes DF, Lie SA, Enersen M, Aastrøm AN, Mustafa K, Leknes KN (2015). Site-specific treatment outcome in smokers following non-surgical and surgical periodontal therapy. J Clin Periodontol.

[32] Berglundh T, Armitage G, Araujo MG, Avila-Ortiz G, Blanco J, Camargo PM, Chen S, Cochran D, Derks J, Figuero E (2018). Peri-implant diseases and conditions: consensus report of workgroup 4 of the 2017 world workshop on the classification of periodontal and peri-implant diseases and conditions. J Clin Periodontol.

[33] Rodriguez MV, Ravidà A, Saleh MHA, Basma HS, Dukka H, Khurshid H, Wang HL, Moreno PG (2022). Is the degree of physiological bone remodeling a predictive factor for peri-implantitis?. J Periodontol.

[34] Papapanou PN, Sanz M, Buduneli N, Dietrich T, Feres M, Fine DH, Flemmig TF, Garcia R, Giannobile WV, Graziani F (2018). Periodontitis: consensus report of workgroup 2 of the 2017 world workshop on the classification of periodontal and peri-implant diseases and conditions. J Clin Periodontol.

[35] Skoglund LA, Vigen EC. Mouthrins against pandemic/epidemic virus – A brief review. Nor Dent J. 2020;130. 10.1038/s41415-020-1485-y.

[36] Hollender L, Rockler B (1980). Radiographic evaluation of osseointegrated implants of the jaws Experimental study of the influence of radiographic techniques on the measurement of the relation between the implant and bone. Dentomaxillofac Radiol.

[37] Romeo E, Lops D, Chiapasco M, Ghisolfi M, Vogel G (2007). Therapy of peri-implantitis with resective surgery. A 3-year clinical trial on rough screw-shaped oral implants. Part II: radiographic outcome. Clin Oral Implants Res.

[38] Raes S, Cosyn J, Noyelle A, Raes F, De Bruyn H (2018). Clinical outcome after 8 to 10 years of immediately restored single implants placed in extraction sockets and healed ridges. Int J Periodontics Restorative Dent.

[39] Barros SP, Williams R, Offenbacher S, Morelli T (2016). Gingival crevicular fluid as a source of biomarkers for periodontitis. Periodontol 2000.

[40] Wewers ME, Lowe NK (1990). A critical review of visual analogue scales in the measurement of clinical phenomena. Res Nurs Health.

[41] Zangrando MSR, Eustachio RR, de Rezende MLR, Sant'ana ACP, Damante CA, Greghi SLA (2021). Clinical and patient-centered outcomes using two types of subepithelial connective tissue grafts: A split-mouth randomized clinical trial. J Periodontol.

[42] Merli M, Bernardelli F, Giulianelli E, Carinci F, Mariotti G, Merli M, Pini-Prato G, Nieri M (2020). Short-term comparison of two non-surgical treatment modalities of peri-implantitis: clinical and microbiological outcomes in a two-factorial randomized controlled trial. J Clin Periodontol.

[43] Monje A, Caballé-Serrano J, Nart J, Peñarrocha D, Wang HL, Rakic M (2018). Diagnostic accuracy of clinical parameters to monitor peri-implant conditions: a matched case-control study. J Periodontol.

[44] Lang NP, Wetzel AC, Stich H, Caffesse RG (1994). Histologic probe penetration in healthy and inflamed peri-implant tissues. Clin Oral Implants Res.

[45] Gerber JA, Tan WC, Balmer TE, Salvi GE, Lang NP (2009). Bleeding on probing and pocket probing depth in relation to probing pressure and mucosal health around oral implants. Clin Oral Implants Res.

[46] Monje A, Vera M, Munoz-Sanz A, Wang HL, Nart J (2021). Suppuration as diagnostic criterium of peri-implantitis. J Periodontol.

[47] Jepsen S, Rühling A, Jepsen K, Ohlenbusch B, Albers HK (1996). Progressive peri-implantitis Incidence and prediction of peri-implant attachment loss. Clin Oral Implants Res.

[48] Carlos Garacioa-Pazmino KS, Hom-Lay Wang: Current Protocols for the Treatment of Peri-implantitis. Clinical Periodontics: Current Oral Health Reports; 2019.

[49] Stein JM, Hammächer C, Michael SS: Combination of ultrasonic decontamination, soft tissue curettage, and submucosal air polishing with povidone-iodine application for non-surgical therapy of peri-implantitis: 12 Month clinical outcomes. J Periodontol 2017. 10.1902/jop.2017.170362 28914597

[50] John G, Sahm N, Becker J, Schwarz F (2015). Nonsurgical treatment of peri-implantitis using an air-abrasive device or mechanical debridement and local application of chlorhexidine. Twelve-month follow-up of a prospective, randomized, controlled clinical study. Clin Oral Investig.

[51] Petković AB, Matić SM, Stamatović NV, Vojvodić DV, Todorović TM, Lazić ZR, Kozomara RJ (2010). Proinflammatory cytokines (IL-1beta and TNF-alpha) and chemokines (IL-8 and MIP-1alpha) as markers of peri-implant tissue condition. Int J Oral Maxillofac Surg.

[52] Esberg A, Isehed C, Holmlund A, Lundberg P (2019). Peri-implant crevicular fluid proteome before and after adjunctive enamel matrix derivative treatment of peri-implantitis. J Clin Periodontol.

[53] Wang HL, Garaicoa-Pazmino C, Collins A, Ong HS, Chudri R, Giannobile WV (2016). Protein biomarkers and microbial profiles in peri-implantitis. Clin Oral Implants Res.

[54] Theodoridis C, Doulkeridou C, Menexes G, Vouros I (2022). Comparison of RANKL and OPG levels in peri-implant crevicular fluid between healthy and diseased peri-implant tissues. a systematic review and meta-analysis. Clin Oral Investig.

[55] Wang Q, Lu H, Zhang L, Yan X, Zhu B, Meng H (2020). Peri-implant mucositis sites with suppuration have higher microbial risk than sites without suppuration. J Periodontol.

[56] Renvert S, Samuelsson E, Lindahl C, Persson GR (2009). Mechanical non-surgical treatment of peri-implantitis: a double-blind randomized longitudinal clinical study. I: clinical results. J Clin Periodontol.

[57] Alqahtani M (2021). Influence of moderate cigarette smoking on the peri-implant clinicoradiographic inflammatory parameters around cement- and screw-retained dental implants. J Oral Implantol.

[58] Hashino E, Kuboniwa M, Alghamdi SA, Yamaguchi M, Yamamoto R, Cho H, Amano A (2013). Erythritol alters microstructure and metabolomic profiles of biofilm composed of Streptococcus gordonii and Porphyromonas gingivalis. Mol Oral Microbiol.

[59] Drago L, Bortolin M, Taschieri S, De Vecchi E, Agrappi S, Del Fabbro M, Francetti L, Mattina R (2017). Erythritol/chlorhexidine combination reduces microbial biofilm and prevents its formation on titanium surfaces in vitro. J Oral Pathol Med.

[60] Matthes R, Duske K, Kebede TG, Pink C, Schlüter R, von Woedtke T, Weltmann KD, Kocher T, Jablonowski L (2017). Osteoblast growth, after cleaning of biofilm-covered titanium discs with air-polishing and cold plasma. J Clin Periodontol.

[61] Mensi M, Cochis A, Sordillo A, Uberti F, Rimondini L. Biofilm Removal and Bacterial Re-Colonization Inhibition of a Novel Erythritol/Chlorhexidine Air-Polishing Powder on Titanium Disks. Materials (Basel). 2018;11(9):1510. 10.3390/ma11091510. 10.3390/ma11091510 PMC6164901 30142888

[62] Schwarz F, Schmucker A, Becker J (2015). Efficacy of alternative or adjunctive measures to conventional treatment of peri-implant mucositis and peri-implantitis: a systematic review and meta-analysis. Int J Implant Dent.

[63] Seymour RA, Blair GS, Wyatt FA (1983). Post-operative dental pain and analgesic efficacy. Part I Br J Oral Surg.

[64] Yang HL, Wu XB, Mao C (2019). Block randomization in clinical trials. Zhonghua Yu Fang Yi Xue Za Zhi.

[65] Garcia-Garcia M, Mir-Mari J, Figueiredo R, Valmaseda-Castellon E (2021). Probing single-tooth dental implants with and without prostheses: a cross-sectional study comparing healthy and peri-implant mucositis sites. J Clin Periodontol.

[66] Koyanagi T, Sakamoto M, Takeuchi Y, Maruyama N, Ohkuma M, Izumi Y (2013). Comprehensive microbiological findings in peri-implantitis and periodontitis. J Clin Periodontol.

